# A mother’s choice: a qualitative study of mothers’ health seeking behaviour for their children with acute diarrhoea

**DOI:** 10.1186/s12913-016-1911-7

**Published:** 2016-11-21

**Authors:** Lucy Cunnama, Ayako Honda

**Affiliations:** Health Economics Unit, School of Public Health and Family Medicine, Faculty of Health Sciences, University of Cape Town, Observatory, Cape Town, 7925 South Africa

**Keywords:** South Africa, Perceived quality, Healthcare access, Child-health, Rural, Traditional practitioners, Qualitative

## Abstract

**Background:**

Diarrhoea presents a considerable health risk to young children and is one of the leading causes of infant mortality. Although proven cost-effective interventions exist, South Africa is yet to reach the Sustainable Development Goals set for the elimination of preventable under-five mortality and water-borne diseases. The rural study area in the Eastern Cape of South Africa continues to have a parallel health system comprising traditional and modern healthcare services. It is in this setting that this study aimed to qualitatively examine the beliefs surrounding and perceived quality of healthcare accessed for children’s acute diarrhoea.

**Methods:**

Purposive sampling was used to select participants for nine focus-group-discussions with mothers of children less than 5 years old and 11 key-informant-interviews with community members and traditional and modern practitioners. The focus-group-discussions and interviews were held to explore the reasons why mothers seek certain types of healthcare for children with diarrhoea. Data was analysed using manual thematic coding methods.

**Results:**

It was found that seeking healthcare from traditional practitioners is deeply ingrained in the culture of the society. People’s beliefs about the causative agents of diarrhoea are at the heart of seeking care from traditional practitioners, often in order to treat supposed supernatural causes. A combination of care-types is acceptable to the community, but not necessarily to modern practitioners, who are concerned about the inclusion of unknown ingredients and harmful substances in some traditional medicines, which could be toxic to children. These factors highlight the complexity of regulating traditional medicine.

**Conclusion:**

South African traditional practitioners can be seen as a valuable human resource, especially as they are culturally accepted in their communities. However due to the variability of practices amongst traditional practitioners and some reluctance on the part of modern practitioners regulation and integration may prove complex.

**Electronic supplementary material:**

The online version of this article (doi:10.1186/s12913-016-1911-7) contains supplementary material, which is available to authorized users.

## Background

Diarrhoeal diseases are an important health problem to address if the Sustainable Development Goals (SDG) are to be achieved globally and, more specifically, SDG 3 and 6, which aim to end preventable under-five mortality by 2030 (SDG 3.2), eliminate water-borne diseases (SDG 3.3) and to provide clean accessible water and sanitation for all (SDG6) [1]. This leads on from the Millennium development goal to reduce the child mortality rate of children under five by two thirds. Approximately 90 % of the 2 million worldwide deaths attributed to pneumonia and diarrhoeal diseases in children under 5 years old in 2010 occurred in Sub-Saharan Africa and South Asia [2].

In South Africa, the under-five mortality rate has decreased from 61 to 45 deaths per 1000 children between 1990 to 2012 [3]. While the country is progressing towards achieving SDG 3, large variations in health status exist between different geographical regions. In fact, in the Eastern Cape of South Africa in 2011 (at the time of the study) the incidence of diarrhoea in children under 5 years of age was 89 per 1000 children [4]. There are relatively cost-effective interventions available to prevent or treat diarrhoeal diseases but the low uptake of these interventions is low [3].

South Africa is in a very unique healthcare situation due to its history of colonial rule, previous apartheid policies and discrimination, post apartheid healthcare reform and longstanding use of traditional practitioners [5]. These factors have produced a pluralistic healthcare system where individuals in society access healthcare from both traditional and modern practitioners [6].

Traditional medicine can be defined as treatment practices that were established in rural areas before the advent of modern medicine and that continue to bear a resemblance to these habitual techniques [7]. Traditional medicine is administered by traditional practitioners, a term which will be used throughout this article to describe a range of practitioners in South Africa who provide traditional medicine in the particular study setting specific to the AmaXhosa culture. These include several different practitioners grouped together such as sangomas, traditional healers and herbalists (other forms of traditional and alternative medicine such as homeopathic medicine have not been included in this study, although they are, at times, mentioned in the included literature). Modern medicine is the term used to describe western, biomedical or mainstream medicine, which is provided by modern practitioners such as doctors, nurses and other clinic and hospital clinical staff. In the context of this paper, modern medicine is provided in up-to-date, South African government-provided healthcare facilities (clinics and hospitals).

Traditional medicine is an important form of healthcare that supports many individuals globally [8]. It is estimated that in low-and middle-income countries, such as South Africa, 80 % of the population utilise traditional medicine [9]. Due to the high utilisation rate, the World Health Organisation (WHO) has found it necessary to carefully integrate and regulate traditional medicine so that it can be provided to society in a safe, efficient and effective way [8].

Although some traditional medicine is ‘tolerated’ by law in South Africa, the public healthcare provided is based on modern medicine [10]. Unlike countries such as Bangladesh, where traditional practitioners belong to a regulatory board and study at a teaching college [11], South African traditional practitioners are currently unregulated and receive no formal training, despite the Traditional Health Practitioners Act that was passed by the South African Government in 2008 [6].

The pattern of utilising traditional practitioners has led to a low uptake of modern medical interventions or delayed treatment of diarrhoeal diseases in children [9, 12]. This treatment approach occurs despite the fact that modern facilities provide free services to children less than 6 years of age.

Timely access to interventions for treatment of diarrhoea can have significant impact on the efficacy of treatment. Access can be conceptualised using a model that considers three dimensions: affordability, availability, and acceptability. Availability refers to geographical convenience, the hours an institution is open and the impact these have on utilisation and the type of services offered by practitioners. Affordability refers to the financial implications of the health seeker’s behaviour and the ability of the individual to pay for services. Acceptability looks at quality of care as perceived by patients and includes the cultural acceptability of services in terms of the individual’s beliefs (both religious and cultural) and the practitioner’s attitudes [13–15]. While there are a relatively large number of studies that address the issues of affordability and availability, little research has been undertaken on the issue of acceptability of services for diarrhoeal diseases in children in the specific context of a plural healthcare system in rural South Africa. As South Africa aims to integrate traditional practitioners into the current healthcare system it is important to better understand the beliefs and the perceptions relating to both modern and traditional healthcare in the rural setting [16].

This study aims to explore the basis of choices made by mothers with children under 5 years old when their children develop diarrheal diseases by looking at the aspect of perceived quality of treatment by traditional and modern medicine. Specifically, the study will examine:

1. Belief systems rooted in the socio-cultural context; and
2. The perceived quality of care provided by traditional practitioners and modern practitioners.

The study also explores the separation between traditional and modern medicines, and possible areas for collaboration between traditional and modern practitioners to improve the health of people in the community.

## Methods

### Study setting

The study took place in the catchment of a rural district hospital in the O.R Tambo district in the Eastern Cape province. The catchment has a population of around 130,000 people and the area is considered deeply rural [17]. The area where the study took place is approximately 90 km from the nearest city of Mthatha [18]. O.R Tambo is one of 18 identified priority health districts in South Africa. The priority health districts have been selected according to maternal and child health and socio-economic indicators and focus is placed on those districts most in need of improvement [19]. The O.R. Tambo district is considered to have some of the poorest health indicators in the country with double the national infant mortality and triple the number deaths for children under 5 years old [20]. The incidence of diarrhoea in the district in 2007 and 2008 was 173.5 per 1000 [21] in comparison to the provincial incidence of 110 per 1000 children in 2009 [4]. The district is home to 183 government-run, modern facilities (hospitals, mobile clinics, community health centres and clinics) that provide modern medicine to people in the district [22]. It is estimated that there are approximately 10,780 traditional practitioners in the Eastern Cape and around 190,000 traditional practitioners practicing in South Africa. This means that there are in the region of 1797 traditional practitioners in each of the 6 districts in the Eastern Cape [23].

Historically, the area where the study was undertaken belongs to the AmaXhosa People, who converse in the language IsiXhosa and continue to practise certain cultural traditions. Prior to the start of the hospital by missionaries in the study area in the 1950’s [17], healthcare was exclusively provided by traditional practitioners, who were believed to converse with the AmaXhosa’s Godlike Supreme Being via their own ancestors to establish the cause of a person’s misfortune or illness [24].

### Data collection

Data was collected in the area, during 3 weeks of January 2011 by the corresponding study author. Data collection methods included both key-informant-interviews and focus-group-discussions with informants and participants being purposively recruited. The focus-group-discussion participants were recruited with the help of modern and traditional practitioners practicing in the area as well as other community members.

There were 11 key-informant-interviews with modern practitioners (three doctors, one nurse and one therapist), two community members (one of whom was a minister) and four traditional practitioners, and nine focus-group-discussions with mothers who have children under 5 years old. Each discussion group had two to nine participants and a total of 32 participants took part. Ages of the women in the discussion groups ranged from 18 to 60 years of age.

Data collection tools included a semi-structured questionnaire for the key-informant-interviews and an agenda for the focus-group-discussions respectively (see Additional file 1). These tools were created using existing literature on traditional and modern medicine, access to healthcare, the South African health system, diarrhoea and child health. Findings from other studies were assimilated to form a list of probable factors, which might influence where a mother would take her child if her child had acute diarrhoea according to the perceived quality of healthcare.

The topics covered in the key-informant-interviews were beliefs (about the causative agents of diarrhoea, spiritual beliefs and belief in traditional medicine), tolerance of patients’ beliefs in traditional medicine and the degree of fit between the beliefs of modern practitioners and those of community members.

The agenda for the focus-group-discussions utilised a scenario about a woman whose child is sick with diarrhoea. The participants were then asked questions regarding the choices she makes and asked what they might do in her situation. The focus-group-discussions covered the agendas concerning the reasons for utilising or not utilising services, participants’ cultural and religious beliefs, their beliefs in the causes of diarrhoeal disease, the perceived responsiveness of the healthcare providers, as well as social pressures.

Key-informant-interviews were held in English where possible, but when it was not possible translation was undertaken on the site by a translator who was fluent in English and IsiXhosa. All focus-group-discussions were held in IsiXhosa and questions and answers were translated on the site by a professional translator. With the consent of participants, an electronic audio recorder was utilised in the interviews and focus-group-discussions.

Mothers who were younger than 18 years of age were excluded as ethical clearance (University of Cape Town, HREC REF 557/2010) was only obtained to question those participants who were legally able to give informed consent to take part in the study. Anonymity is maintained in the reported results.

### Data analysis

A qualitative exploratory approach was used for data analysis. Both inductive and deductive processes were utilised to analyse the data. Based on a review of literature, a pre-determined coding scheme was developed and used to analyse the data, however other important themes identified during the process of data collection and analysis were also explored in order to examine issues that had not been anticipated prior to the collection of data.

The electronic audio files were transcribed word for word into English. The quality of translation provided during the interviews and focus group discussions was ensured by the primary investigator verifying the translations of IsiXhosa to English in the transcriptions.

Coding was done manually, by firstly identifying recurrent ideas, grouping these systematically into broader themes, reducing the themes into concepts and then seeing how they applied to the framework objectives developed prior to the study.

## Results

To understand the basis of mothers’ choices about healthcare for children suffering from diarrhoea, this section first presents the common behaviours of mothers when choosing healthcare providers for children suffering from a diarrhoeal disease; followed by mothers’ beliefs about the cause of diarrhoeal diseases, and their experience of both traditional and modern practitioners. Finally, the potential for collaboration between modern and traditional practitioners is explored.

### Common behaviour of mothers choosing healthcare providers when their children develop diarrheal diseases

At the site of the study, mothers typically use a mixed care approach when seeking healthcare for children with diarrhoea. Mothers choose whether they will take their children to traditional practitioners or modern practitioners depending on the immediate situation and quite often seek care from both traditional and modern practitioners:

“Oh, I just decide…if I think ‘today I must go to the hospital,’ I just go to the hospital. If I think ‘today I must go to a traditional healer,’ [I] just go to the traditional healer.” (Focus Group Discussion 1)

Mothers make up their minds by looking at their children’s level of suffering, symptoms and their own judgement about what traditional and modern medicine practitioners can do:

“There are diseases that you hear that the person is sick…and you know whether that is suited for a western or an African way of treatment.” (Community Member 1)

“It depends on how the person is sick because even if you are a traditional healer and you are a good one, there are those things…that [you] can’t even heal. And even if you can go to hospital there are those diseases that they cannot heal [in] hospital, so you can end up going to [a] traditional healer.” (Traditional Practitioner 1)

Some mothers take their children to the nearest available practitioner. Given that there is limited availability of modern medicine facilities in rural South Africa, including at the study site, when children are very unwell, it is often easier for mothers to obtain treatment for children from traditional practitioners:

“You [are] just desperate, you need help so you don’t know where you are going to get the help you need so you go to the nearest place that you hope they are going to help you.” (Focus Group Discussion 2)

The mothers’ behaviour of ‘hopping’ between traditional and modern practitioners can end in tragic consequences. The following account, describing a series of choices made by a mother, illustrates the complexity of decision-making by mothers about their children’s healthcare. Furthermore, the course of action taken by the mother demonstrates how traditional and modern healthcare are used in tandem.

“Unfortunately my child passed away there at hospital because I tried to take her to the traditionals [traditional practitioner] first. I took her to the hospital later. It was [too] late for her. [When it started] she was having *ipleyiti* and she was vomiting. I took her here…to the clinic. Then I went and got medicine for *ipleyiti*. So that medicine did help, also I took the child to the doctor [general practitioner in a nearby town approximately 60 km away]. So they [the general practitioner] sent me to the hospital…he didn’t even take the money, just sent me to the hospital. At the hospital they didn’t give me much care, because I used that [herbal] medicine…They gave her [the child] the drip but after that it was already too late [the baby had passed away].” (Focus Group Discussion 6)

### Mothers’ belief systems and healthcare services provided by modern facilities

The main area of contention between mothers and modern facilities is the denial of mothers’ belief systems by modern practitioners (or rather the health services provided at modern facilities do not fit with what mothers believe). This discrepancy means there is a narrow platform for communication and misunderstanding can easily arise.

“…if you first take the child to a traditional healer, so when you take the child to the hospital they can see… when they check the child they can see that you’ve given the child something herbal…They think it’s a bad thing.” (Focus Group Discussion 6)

“…they become rude, they become arrogant… they can judge me by telling me about these [indicating white bead bangles on wrists and ankles], maybe this is not good. And I know why I have got these things, but the doctors don’t like them, don’t like these things.” (Traditional Practitioner 4)

However, while mothers’ choices in relation to healthcare for their children are strongly influenced by their cultural beliefs, they actually do not mind taking their children to modern facilities when such visits are required and they are considered to be able to provide treatment to cure their children’s diarrhoea.

“We don’t take our children to traditional healers anymore…we take our children to the clinic. I believe that the traditional healer can help and the hospital also can help. So we take our children to the clinics.” (Focus Group Discussion 6)

Mothers’ choices about seeking care at modern facilities have been influenced by the increased number of modern health facilities available in rural South Africa, people’s increased experience of, and familiarity with, modern facilities, and an improvement in people’s perceptions of modern facilities.

“…A while ago there were few clinics. Clinics and hospitals were scarce so…that is why most of the time…they [the community] were going to the sangomas [traditional practitioners]…Now there are more clinics and…most of the time they take their children to the hospitals and even if they are sick [the adults] they go to the hospitals, unlike before, when there were no clinics and hospitals.” (Focus Group Discussion 8)

“…it’s a good hospital…nowadays it’s not like before when our mothers used to go there. Like now, everything is done well there. It’s the right hospital.” (Focus Group Discussion 7)

Also, mothers appear to be attracted by some forms of healthcare, such as the intravenous drips that children can receive at modern facilities when they develop diarrheal diseases:

“…take the child to the hospital because, even if you can take the child to a sangoma, she can’t help you if that child, especially if the child has got diarrhoea. Because the sangoma doesn’t have [a] drip…” (Focus Group Discussion 2)

However, some respondents said that they had been treated and discharged from the hospital without the hospital staff explaining the problem to them, but it could be that the understanding of the underlying cause differed between the mother and hospital staff:

“And sometimes at hospital they don’t know…and sometimes if the child doesn’t get help at hospital… I can take the child to the traditional healer because sometimes my child has got *inxeba* of which at hospitals they can’t help, they don’t know about that.” (Focus Group Discussion 1)

### Mothers’ beliefs about the cause of diarrheal diseases and their choice of traditional practitioners as healthcare providers for their children

Mothers and practitioners interviewed indicated that when mothers choose healthcare providers for the treatment of children with diarrheal diseases, the decision is based on the mothers’ beliefs about the causal agents of diarrheal disease (i.e. the involvement of curses) that are deeply rooted in their culture and society, and supported by previous experience of seeking care from traditional and modern practitioners.

The community examined in the study attributes many of the causes of diarrhoea to curses, witchcraft and evil spirits, and traditional practitioners are considered to be able to treat these causes:

“There is help from a traditional healer and there is help from the hospital so you don’t know where your child is going to get help. And there are things that sangoma can do and the hospital cannot do and there are things that the hospital can do that a sangoma cannot do. Sometimes you believe the child is being betwitched so that is why most of the time the sangoma is going to give you an emema.” (Focus Group Discussion 2)

“All these things happen because of bad spirits. *Ibala,* 1 maybe there’s something that you…when you put your foot on…something like that can happen. And *inxeba* 2 also is a bad spirit also, it’s a curse.” (Focus Group Discussion 4)

In addition, some mothers prefer traditional practitioners to modern practitioners as a result of their experience of the care that they have received from traditional practitioners:

“…the reason why people like to go to the traditional healers is they give full care to the people, they care a lot about the people and there are things that they know that you can’t go to the hospital having this thing.” (Focus Group Discussion 9)

However, depending on the mother’s beliefs or previous experience she may also seek care at a modern facility.

“I go to the clinic first always because sometimes my child can get diarrhoea because of the heat. So there at hospital they know that, so they can tell you if it’s heat [causing diarrhoea].” (Focus Group Discussion 1)

“I think quite often if they have had a positive experience before then they are more likely to seek hospital care again. Or if they have gone to a traditional healer and it has not worked they might come [to the hospital].” (Modern Practitioner 1)

Mothers face a certain degree of socio-cultural pressure from family members and/or neighbours about their choice of healthcare services for their children but it is not a strong driver in determining mothers’ healthcare-seeking behaviour for their children. Mothers try to choose the best option for their children based on their own belief systems, past experience and their perception of the health status of their children.

“Because every mother wants to do what’s best for the child and they believe it’s good and helpful.” (Modern Practitioner 3)Potential for cooperation between modern practitioners and traditional practitioners

A number of traditional practitioners and people in the community are open to accessing the health services provided by modern facilities, and little negative was said on the subject.

“It depends on your beliefs as the mother of the baby. There is nothing wrong with…taking your child to hospital. And there is nothing wrong with traditional medicine.” (Traditional Practitioner 1)

Conversely, modern practitioners are a little more resistant to working with traditional practitioners due to differences in the nature of care and the philosophies associated with the two types of healthcare services.

“… originally I’m from this town. I was born in a rural area on the other side. Then I left I went to another town to stay there, because of family issues and all that. But originally I was born and bred and grew up here…My personal point of view, I totally disagree and I’m against it [traditional medicine]. With the knowledge that a child has got a gut that is not well developed and since it is not well developed it is unable to function and metabolise everything that gets in there. That is why the first 6 months it’s supposed to be fed full breastfeeding, because the gut is not prepared to digest and to metabolise. And so that is why I’m completely against [using traditional medicine for children] myself, as a person.” (Modern Practitioner 5)

Although traditional medicine is not necessarily accepted by modern practitioners, modern practitioners are aware that they may not understand all the aspects of mothers’ decision-making.

“But it is naïve to think because of what we see at the hospital we can actually judge the entire function of society here.” (Modern Practitioner 1)

“I feel that I’m very…um…judgmental and I often have to take a step back and realize I am being judgmental because I realize that my understanding of disease isn’t complete either, that my western medicine, economic, social view, doesn’t…take into account the…spiritual side and the cultural side of things and I don’t understand that and so I am quick to judge.” (Modern Practitioner 2)

Modern practitioners also express concerns about some of the substances used in the medicines given by traditional practitioners to patients (such as detergents).

“I mean there are lots of things that have just like detergents and some things that are completely ridiculous that I can’t…I just don’t understand why they give them at all… It’s so difficult. The ones that are the worst are the tiny ones and you can actually see…the detergent coming out of their noses in bubbles and you smell and it’s so obvious. That is very hard to see and not pass a very quick judgment which, in saner moments, is not usually the best idea.” (Modern Practitioner 1)

“That’s what makes me scared - it’s so unregulated. …I mean if everybody did the same thing, it would be easier to form a relationship that worked where we could say ‘well we’ll look after these things and please would you look after these things’. You know, ‘please, please don’t give tiny babies plate^3^ [*ipleyiti*] medicine or give the traditional plate medicine [and] not this dreadful jik [a brand name of a commercial cleaning agent with bleach as a main ingredient] containing substance’…But everybody does things slightly differently. Then it’s hard kind of… It’s hard to…make like a…referral system …when everybody does things just a little bit differently.” (Modern Practitioner 1)

Furthermore, taking children to traditional practitioners before seeking care from modern facilities may result in delays in obtaining the care necessary for children with diarrheal diseases to successfully recover.

“As far as using traditional medicine to the babies, because we have seen a lot of babies dying, in fact, we still see them every now and then that the mother they take the baby to a traditional healers.” (Modern Practitioner 5)

Although there is some reluctance to work together, there is feeling that modern practitioners would like to learn more about traditional practitioners and their patients’ beliefs:

“I think there is definitely…a role for us working together…I don’t actually know exactly how a sangoma would deal with those sorts of issues but…it’s something we could explore” (Modern Practitioner 2)

“I don’t think that we’ve listened enough and heard enough and I don’t think we talk with our patients enough. I think we are quick to judge but it’s because we have seen so much damage from what they [the community] believe.” (Modern Practitioner 2)

## Discussion

The study explores the choices made by mothers in rural Eastern Cape about healthcare services for their children when they develop diarrheal diseases, identifying patterns in mothers’ health-seeking behaviour for their children and the reasons behind their choices. The study reveals that: beliefs about the causative agents of diarrhoea and traditional medicine and the observed effects of traditional medicine are central to the decision-making process of the mothers of children suffering from diarrhoea; mixed healthcare (both traditional and modern healthcare) is common in the area; medical technologies are seen as a draw card to encourage people to attend modern facilities however, there is often an absence of medical knowledge about the rationale for the use of modern medicine; some harmful substances, such as, but not limited to, detergents (for their cleansing properties), are used as ingredients in some traditional practitioner treatments; social pressure does not play a prominent role in decision-making; and although seeking care from both traditional practitioners and modern facilities is acceptable to the community and some traditional practitioners, some modern practitioners may be slightly more resistant to accepting the practices involved in traditional medicine and patients that seek traditional healthcare.

The discussion of the study results attempts to focus on understanding mothers’ decisions about healthcare providers for children with diarrheal diseases; and how healthcare can be improved in a context where two types of healthcare providers (i.e. modern and traditional) operate in parallel and people seek healthcare services from both types of providers. It is important to note that the traditional medicine referred to in this study is the traditional medicine that the authors observed being practised in rural Eastern Cape and does not include other types of traditional medicine.

The main finding of this study is that the belief systems of the community in rural Eastern Cape dictate how caregivers access child healthcare. Seeking healthcare from traditional practitioners is deeply ingrained in the AmaXhosa culture and the choice of traditional practitioners by mothers seeking care for their children is rooted in cultural beliefs. Traditional practitioners are perceived as being able to treat ‘traditional illnesses’ and traditional illnesses arise as a result of supernatural causes. Modern facilities are believed to be unable to treat ‘traditional illnesses’. This result corresponds to the limited literature available on treatment of childhood disease with traditional medicine in South Africa [12, 25]. The most common reason for seeking healthcare from traditional practitioners is treatment for illnesses that people believe are associated with witchcraft, spirits and ancestors, and which people believe modern facilities are powerless to treat [12]. The explanation that modern practitioners provide for “traditional illnesses” does not meet with the expectations of people in the community [25]; and people’s belief or trust in the treatment given by traditional practitioners is the main reason that people choose traditional practitioners as their healthcare providers [12, 26]. Due to the strong belief in traditional medicine and observed effects of traditional treatments by members of the community, modern practitioners are seen as unable to adequately explain and treat health problems [12]. Furthermore, staff in modern facilities are often perceived not to understand the underlying cause of illness. Furthermore, caregivers do not always understand the modern medical theory of illness, its causative agents and the appropriate treatment.

This study supports work by Friend-du Preez et al. [12] examining the treatment of childhood illness in urban South Africa, in that: symptoms often dictate which type of practitioner a child is taken to see; efficacy of previous treatment adds to the trust in traditional practitioners and the continued use of traditional medicine; the use of a medical technology, such as a drip, attracts people to use hospitals in conjunction with traditional practitioners, but not all mothers are aware of the reasons for use of an intravenous drip. Many mothers do not mind seeking care from modern practitioners, but their choice is based on their previous experience of modern medicine and the attraction of a particular type of treatment.

Interestingly, and in contrast to this study’s findings, Friend-du Preez et al.[12] found that the vulnerability of small children was seen as a reason to seek care from traditional practitioners, as caregivers felt a strong need to protect children from bad spirits. The current study found that some community members feel that an infant’s vulnerability is a good reason not to administer unknown dosages of traditional medicine, and would rather take a child to a clinic or hospital when the child is ill. Also, social pressure was not found to be as important in influencing mothers’ health seeking behaviour as belief about the causative agents of diarrhoea and traditional medicine.

In the past 15 years, efforts have been made to improve access to modern healthcare in South Africa, including the introduction of a clinic building program, free primary healthcare and exemptions from hospital fees for the poor [27]. While there has been a decline in the use of traditional medicine [28], the majority of the rural population still use traditional medicine, either exclusively or in combination with modern medicine [9]. Indeed, pluralistic care seems to be common in the studied area, with multiple healthcare providers accessed even for one case of illness. Mothers appear to move between modern and traditional practitioners when they seek care for children with diarrhoeal diseases. The treatment mix chosen by mothers is often based on the perceived severity of symptoms in children and their knowledge of what modern and traditional practitioners can do, as well as the availability of practitioners in their area.

The WHO has suggested that it is important to recognise the alternative means of treating health problems when these means are socially and culturally imbedded in the context, and the complementary use of both modern and traditional medicines should be promoted [29]. In addition, the WHO suggests that traditional medicine should be considered as a source of healthcare services, particularly in rural or disadvantaged areas where the availability of health services is extremely limited due to geographical and socio-economic conditions. Several countries, including China, the Republic of Korea and Vietnam, have achieved full integration of local traditional medicine into their health systems, while other countries, such as Switzerland, are partially integrated [8]. In the state of Tamil Nadu in India, the government has made a concerted effort to incorporate local traditional medicine into its primary healthcare services. Support of traditional practitioners’ services has been provided by promoting research into traditional medicine [11].

In South Africa, there are few or no policy guidelines or legal frameworks on how traditional medicine can operate and/or co-exist with modern medicine in the health system. Consequently, a wide variation in the provision and types of traditional medicine exist, and the choice of healthcare providers is left to people who are likely to lack sufficient technical knowledge to make informed decisions about appropriate healthcare. Currently there are gaps in knowledge and understanding of the roles and practices of modern and traditional practitioners, by both these groups and the mothers in the community.

The absence of a framework outlining how traditional and modern practitioners can operate together in the health system, the lack of guidelines and/or sufficient information to guide people’s healthcare choices, and pluralistic healthcare mechanisms resulting in patients ‘hopping’ between modern and traditional providers, which can all result in delays in treatment, or mixed treatment, leading to inconsistent and/or incomplete medical care [12, 30]. In addition, there have been alarming reports that some forms of traditional medicine contain harmful substances, which can further complicate disease or injury [12, 31].

The authors’ view of the study area’s traditional practitioners and combining different types of traditional practitioners into one group for the study (for practical reasons) may demonstrate a limited understanding of the structure of traditional medicine in South Africa. It must also be mentioned that traditional medicine in other contexts may be different, and may have other outcomes and cultural perspectives than those found in this study. A potential limitation of the participant sampling strategy used, is that the results from this study may not capture the full spectrum of opinions and may not be representative of the whole community.

Since the collection of data in 2011, the South African National Department of Health has been making efforts to formalise and professionalise traditional health providers through the development and implementation of the Traditional Health Practitioners Act [16, 32]. However, the implementation of Act is proceeding very slowly and the context within which traditional health practitioners operate remains unchanged from 2011.

Future studies on the acceptability of various types of healthcare in the Eastern Cape could highlight common issues relating to the integration of modern medicine in rural areas and facilitate a more holistic and culturally appropriate health system. Further research on the regulation of traditional practitioners in the provision of safe and effective treatments is necessary.

## Recommendations

This research found that there is limited collaboration and communication between modern and traditional healers. However if healthcare for rural communities in South Africa is to improve, potential links between the practice of modern and traditional medicine should be considered. A number of issues must be addressed from the perspectives of modern medicine practitioners, traditional practitioners and patients if the integration of the two treatment types is to be achieved. Firstly, in order to better serve the community, modern practitioners need to become more aware of the cultural beliefs and understanding of illness held by people in the community. Secondly, the community needs to be informed about the modern medical understanding of the causes and treatments of diarrhoea. This requires improvement in health education systems, particularly those aimed at women, who are the primary caregivers for children. Finally, the role of traditional practitioners needs to be respected, guided, regulated and possibly restricted if the healthcare they provide is hazardous to small children. The research found that there is a high degree of variability of practice amongst traditional practitioners, which may make setting up a referral system difficult. However traditional practitioners could be asked to work alongside modern medicine but within a limited area, using a system of referral to regulated, modern facilities when appropriate [25, 30].

This study revealed a high level of community acceptance of traditional practitioners. Other countries, such as Bangladesh and India, have successfully utilised ‘lay’ doctors [11], trained in basic healthcare, to improve the dispersion of healthcare information, an approach which could be beneficial in South Africa and traditional practitioners could be used as a valuable human resource with significant understanding of the cultural issues in their communities.

Although traditional medicine appears to be highly accepted in the community studied, it is not seen as acceptable by all modern practitioners. While there is a need to increase the understanding of traditional medicine by modern medicine practitioners, the practice of including some harmful substances in the traditional treatments given to children is a valid concern. Consequently, the Government should provide clear guidelines and a regulatory framework for traditional medicine so that traditional practitioners provide safe, efficacious treatment, which satisfies a set of standards that are equal in rigor to modern medical principles and are in accordance with patients’ human rights [33]. The process of establishing a policy framework on traditional medicine entails the creation of registration mechanisms, regulatory structure, mechanisms for training and accreditation. This requires commitment from both national and provincial governments. It is important that traditional practitioners are not exploited in the process of regulating treatment practices and medicine, and care is needed to protect the intellectual property rights of traditional practitioners if the pharmaceuticals they use are proved to be safe and efficacious.

The study identified that community members have a lack of understanding of the causes of illness and treatment options. In some cases this could result in mothers not questioning the treatment given by either traditional or modern practitioners. Improved education, with an emphasis on female literacy and health awareness, is necessary so that the rural population is better equipped to make informed choices about their own healthcare. Currently, patients are bridging two parallel healthcare systems and seeking healthcare services from both types of healthcare providers. Consequently, empowerment of the community through the provision of health information is critical, requiring collaboration between, and significant effort from, both health administrators and community-based organisations.

Communication channels need to open between traditional and modern practitioners so that a common understanding can be reached. Modern practitioners could meet with the community members to learn how to develop more culturally appropriate services. However, as time and other resources are limited for both modern and traditional practitioners, involvement of government bodies, such as the Provincial Department of Health, may be necessary.

## Conclusions

The acceptability of healthcare is a complex issue. Perhaps even more complex and intriguing are the reasons that mothers make the healthcare choices that they do. This study has revealed that, in general, the community being examined continues to have a profound belief in traditional medicine, although some members have a preference for seeking care from modern medicine practitioners. Mothers seem to have clear reasons for choosing a particular form of healthcare for their children, however the course of action chosen does not always result in the desired effect of successfully treating children with diarrhoeal disease.

It is recommended that appropriate guidelines and a legal framework needs to be established for traditional practitioners to provide safe, efficacious treatment to patients in accordance with patients’ human rights. As regulation is a resource intensive and time consuming process, traditional and modern practitioners should commence a dialogue aimed at developing a mutual understanding, and people in the community, who access both types of healthcare providers, need to be empowered with more knowledge on health and healthcare in order to make appropriate decisions about their own health and health of their children.

## Additional file

Additional file 1: Information for key informant interviews and focus group discussion. This supplementary information includes informed consent (verbal and written), as well as outlines for the key informant interviews and focus group discussions. (DOCX 21 kb)

## Abbreviations

SDG: Sustainable Development Goals
WHO: World Health Organisation

## Footnotes

1 ‘Ibala’ is an illness that is caused by a bad spirit before the child is born for instance if the pregnant mother was cursed by a bad spirit. The English translation is ‘colour’ in reference to the red mark on the child’s neck that is sometimes attributed to ‘ibala’.

2 ‘Inxeba’ is described as a bad spirit that affects a baby after they are born. The translation to English is ‘wound’.

3 ‘Plate’ or ‘ipleyiti’ is a ‘traditional illness’ described as diarrhoea, which is attributed to witchcraft or ‘pollution’, from breast milk or during pregnancy or during the birthing process, which then needs to be cleaned out of the child’s system with plate medicine.
