# Supplementary material for: A mother’s choice: a qualitative study of mothers’ health seeking behaviour for their children with acute diarrhoea
Source: BMC Health Serv Res. 2016 Nov 21;16:669. doi: 10.1186/s12913-016-1911-7 (PMC5117598; doi:10.1186/s12913-016-1911-7)
Supplement: Additional file 1: — Information for key informant interviews and focus group discussion. This supplementary information includes informed consent (verbal and written), as well as outlines for the key informant interviews and focus group discussions. (DOCX 21 kb) [file 12913_2016_1911_MOESM1_ESM.docx]

**Additional file 1**

**Information for key informant interviews and focus group discussion**

***Written informed consent for key informant interviews***

Acceptability of access to child health care, in the Eastern Cape of South Africa.

This study is conducted by Lucy Cunnama (together with Ayako Honda) from the University of Cape Town, Health Economics Unit, Masters in Public Health Department for a Masters Dissertation. It may be published in the future.

The purpose of the study is to investigate the acceptability of healthcare in the area. The significance of this study is to see what affects the acceptability of accessing healthcare in terms of access to child health care services, in particular for the treatment of diarrhoeal disease.

Key informants from the area who give informed consent will be able to take part in the study. Questions will be asked and the answers will be recorded using an electronic audio recorder. A translator will assist in translation if key informants speak isiXhosa. These interviews will then be transcribed and written in English. Interviews will begin on the 10^th^ January 2011 and will cease on the 29^th^ January 2011. The interviews will take approximately one hour. There will be no follow up interview.

Each subject's transcribed interview will be encoded to ensure confidentiality and the participants may withdraw from the study at any stage. Feedback of results will be available to participants on request. There will be no risks, benefits nor cost involved for the participants in this study. There is no insurance cover for participants in this study should harm come to any subjects. Ethical approval for this study is secured from The Faculty of Health Sciences Human Research Ethics Committee. All the research will adhere to the Declaration of Helsinki (2008).

If there are any further questions with regards to the study feel free to contact Lucy Cunnama at lucy.cunnama@uct.ac.za or cell number xxx. In additional, participants can contact the Human Research Ethics Committee (contact xxx) if there are any questions regarding their Rights and welfare as research subjects.

***Informed Consent Form***

University of Cape Town, Health Economics Unit in the School of Public Health and Family Medicine, Faculty of Health Sciences.

I .................. have read (or had read to me by ..................) the information sheet. I understand what is required of me and I have had all my questions answered. I do not feel that I am forced to take part in this study and I am doing so of my own free will. I know that I can withdraw at any time if I so wish without any repercussions.

Signed:

Participant .................. Date and place ..................

Researcher .................. Date and place ..................

Witness .................. Date and place ..................

***Key Informant interview outline***

Key Informant interview will last approximately 1 hour.

Key informant title or role: e.g. Doctor ...................

Questions about acceptability of child health care: approximately 50 minutes

*People's belief in or the perceived effectiveness of traditional medicine/ hospitals.*

1. Why do mothers seek care from a traditional practitioner when their children have diarrhoea?
2. Why do you think a mother whose child has diarrhoea would go to a hospital for treatment?

*People's belief in or perception of causative agents and appropriate treatment of diarrhoea.*

1. Do you think that mothers who take their children to a traditional practitioner/ hospital when they have diarrhoea, have a different understanding of the causative agents and appropriate treatment of diarrhoea, compared to you?
2. Do you/ traditional practitioners /doctors and nurses give information to mothers regarding cleanliness, the mixing of a rehydration solution and encourage mothers to breast feed exclusively in the infant's first 6 months of life?

*Existing social pressure regarding certain types of health care services and perceived responsiveness of traditional and formal health care providers*

1. Do you think your/ traditional practitioners / nurses and doctors' beliefs influence the way the patients are treated?

*Attitudes (tolerance) of health care workers/ traditional practitioners towards patient’s beliefs (regarding the effectiveness of traditional medicine and causative agents and appropriate diarrhoea treatment).*

1. What do you think about using traditional medicine for children?
2. Do your cultural and religious beliefs oppose those who seek care from a traditional practitioner/ hospital?
3. Do you consider yourself/ traditional practitioners/ doctors and nurses tolerant of patient's beliefs?

Conclusion: approximately 5 minutes.

Ask the key informant if he has anything more that he/she would like to say. Thank him/her for participating and give him the thank you letter.

***Standardized Introduction to Focus Group Participants***

My name is Lucy Cunnama and I am studying for a Masters in Public Health at the University of Cape Town. I am doing a project to try to find out what people around this area think about traditional medicine and the hospital and why you want to or don't want to go to hospital or a traditional healer.

I do not work for the government and I have no money for you. I am just asking you to talk with me in this group. If you don't want to talk you don't have to stay and you don't have to answer my questions. But it would help me if you talked with me so that I can understand. You are free to leave at any stage. When I write my project I will not write down anyone's names. I will just write that I was told these things by a group of people from this area. This project might help people to understand better, why people go to the hospital or traditional healer or don't go to the hospital or traditional healers. I am not promising you that anything will change. Do you agree to talk with me?

***Focus Group Outline***

Group discussion will last 1-2 hours.

Introduction (Standardized Introduction to Focus Group Participants).

Group task one (ice breaker): Communication.

Line up oldest to youngest.

Group task two (ice breaker): Trust.

Two people blindfolded, one feeds the other person a sweet.

Ideas for group discussion: approximately 50 minutes.

Outline scenario: *A mother wakes up in the night and her child has diarrhoea and is very sick. She holds the child and then puts him down to sleep. In the morning he still has diarrhoea and is very weak. She takes him to the nearby traditional healer.*

1. Why did the mother go to the traditional healer? Prompt if no response: Did someone tell her to go there? (People's belief in or the perceived effectiveness of traditional medicine).

2. What could have caused the diarrhoea in her child? (People's belief in or perception of causative agents and appropriate treatment of diarrhoea).

3. What does the traditional healer do for the child and does he explain the problem? (Perceived responsiveness of traditional and formal health care providers).

4. Do you believe it works? (People's belief in or perception of causative agents and appropriate treatment of diarrhoea).

5. Why did she not take him to the clinic? (Attitudes of formal health care workers towards patients beliefs).

*The traditional healer gives the child an enema and the mother takes the child home. The diarrhoea gets much worse and now there are no tears when her child is crying. She takes him to hospital.*

6. Why did she go to hospital? Prompt if no response: Did someone tell her to go there? (People's belief in or the perceived effectiveness of traditional medicine and existing social pressure regarding certain types of health care services).

7. If your child gets sick where do you go?

8. Does going to hospital fit with your cultural or religious beliefs? (People's belief in or perception of causative agents and appropriate treatment of diarrhoea).

9. Does the person that helps your child explain the problem to you? (Perceived responsiveness of traditional and formal health care providers).

*The child is given a drip and some strong medicine and is much better. The*

*child and mother go home. The next week the child again gets diarrhoea.*

10. Where does she take the child this time? (Existing social pressure regarding certain types of health care services and perceived responsiveness of traditional and formal health care providers).

11. Why does she take him there? (People's belief in or perception of causative agents and appropriate treatment of diarrhoea and attitudes of formal health care workers towards patients beliefs).

Conclusion: approximately 10 minutes.

Ask each participant if they have anything more that they would like to say. Thank the group for participating.

Thank you very much, without your help I could not have done my project. Thank you for telling me your opinions and for talking to me. I have learnt a lot from you and hopefully other people can also learn from what you have told me. I may send my project to a journal so that other people will be able to learn from this talk and the other talks I have had with other people around this area. But I will not write your names down or tell people who told me what.
